# The "survival of the fittest" bias: how excluding prehospital deaths distorts the impact of transport time in rural trauma

**DOI:** 10.1186/s13049-026-01570-z

**Published:** 2026-02-16

**Authors:** Yingzhe Zhang

**Affiliations:** Department of Pharmacy, Shaoxing Central Hospital, Shaoxing, China

**Dear Editors,**

We read with great interest the study by Lundberg et al. examining prehospital time intervals and 30-day mortality across population density levels in Sweden using the Swedish Trauma Registry (SweTrau) [1]. The authors should be commended for leveraging a nationwide registry, transparently reporting component time intervals, and attempting multivariable adjustment that includes injury severity and prehospital physician involvement—features that make the work highly relevant to prehospital trauma system evaluation [1].

However, we are concerned that the study’s design may be vulnerable to survivorship bias due to left truncation, which could bias estimates toward a null association between prehospital time and mortality. In trauma, early deaths from time-critical injuries may occur well before the traditional “golden hour” [2]. Left truncation arises when individuals who would otherwise meet the target population definition are unobserved because they do not “survive” to become observable at study entry [3]. Moreover, a substantial proportion of trauma deaths occur before hospital arrival [4]. In SweTrau, inclusion requires hospital reception/registration, and patients who die at the scene or are dead on arrival are not captured [1]. If rural transport times are longer, the most severely injured patients may be disproportionately lost before registry entry, creating an apparent “healthier survivor” rural cohort compared with urban areas where proximity increases the likelihood that critically injured patients reach hospital and are recorded. This selection mechanism could plausibly attenuate or even reverse observed associations between transport time and mortality.

Second, restricting analyses to cases with complete prehospital time data may introduce selection bias if missingness is associated with patient acuity or resuscitation intensity (i.e., potentially not missing at random). In emergency settings, time-stamp documentation is often least reliable in the most time-critical cases, making complete-case analysis potentially non-representative. We therefore encourage transparent reporting of the extent and patterns of missingness, alongside consideration of approaches such as multiple imputation with sensitivity analyses under plausible missing-data assumptions [5].

Finally, the observed association between prehospital physician involvement and lower mortality may reflect residual confounding or system-level differences, including variation in termination-of-resuscitation and on-scene pronouncement practices that could influence whether fatalities are transported and subsequently captured in hospital-based registries [1, 6]. We therefore suggest cautious interpretation of this covariate effect unless additional analyses can better account for such structural differences.

To strengthen causal interpretability, we invite the authors to consider sensitivity analyses that incorporate prehospital fatalities (e.g., linkage with the Swedish Cause of Death Registry) and to report robustness checks for missing time data (e.g., multiple imputation with sensitivity analyses under plausible MNAR scenarios). Collectively, these steps could help quantify the potential magnitude and direction of selection and missing-data biases, and thereby better inform policy decisions regarding rural transport resources and HEMS prioritization.

## Data Availability

No datasets were generated or analysed during the current study.

## Abbreviations

HEMS: Helicopter emergency medical service
MNAR: Missing not at random
OR: Odds ratio
SweTrau: Swedish Trauma Registry
